# African Lineage *Brucella melitensis* Isolates from Omani Livestock

**DOI:** 10.3389/fmicb.2017.02702

**Published:** 2018-01-15

**Authors:** Jeffrey T. Foster, Faith M. Walker, Brandy D. Rannals, M. Hammad Hussain, Kevin P. Drees, Rebekah V. Tiller, Alex R. Hoffmaster, Abdulmajeed Al-Rawahi, Paul Keim, Muhammad Saqib

**Affiliations:** ^1^Department of Molecular, Cellular, and Biomedical Sciences, University of New Hampshire, Durham, NH, United States; ^2^Pathogen and Microbiome Institute, Northern Arizona University, Flagstaff, AZ, United States; ^3^Department of Clinical Medicine and Surgery, University of Agriculture, Faisalabad, Pakistan; ^4^Animal Health Research Center, Ministry of Agriculture and Fisheries, Muscat, Oman; ^5^National Center for Emerging and Zoonotic Infectious Diseases, Centers for Disease Control, Atlanta, GA, United States

**Keywords:** brucellosis, *Brucella melitensis*, camels, Oman, MLVA, SNP genotyping

## Abstract

Brucellosis is a common livestock disease in the Middle East and North Africa, but remains poorly described in the region both genetically and epidemiologically. Traditionally found in goats and sheep, *Brucella melitensis* is increasingly recognized as infecting camels. Most studies of brucellosis in camels to date have focused on serological surveys, providing only limited understanding of the molecular epidemiology of circulating strains. We genotyped *B. melitensis* isolates from Omani camels using whole genome SNP assays and VNTRs to provide context for regional brucellosis cases. We identified a lineage of *B. melitensis* circulating in camels as well as in goats, sheep, and cattle in Oman. This lineage is genetically distinct from most genotypes from the Arabian Peninsula and from isolates from much of the rest of the Middle East. We then developed diagnostic assays that rapidly identify strains from this lineage. In analyses of genotypes from throughout the region, Omani isolates were genetically most closely related to strains from brucellosis cases in humans and livestock in North Africa. Our findings suggest an African origin for *B. melitensis* in Oman that has likely occurred through the trade of infected livestock. Moreover, African lineages of *B. melitensis* appear to be undersampled and consequently are underrepresented in genetic databases for *Brucella*. As we begin to more fully understand global genomic diversity of *B. melitensis*, finding and characterizing these unique but widespread lineages is essential. We predict that increased sampling of humans and livestock in Africa will reveal little known diversity in this important zoonotic pathogen.

## Introduction

*Brucella melitensis* is a ubiquitous and common pathogen of goats and sheep worldwide (Seleem et al., 2010; Moreno, 2014). This pathogen was first identified in Malta by David Bruce in 1887, with subsequent discovery of the role of contaminated goat's milk for brucellosis infections in humans by Themistocles Zammit in 1905 (Vassallo, 1996; Wyatt, 2005). Despite apparent host specificity of *B. melitensis* to caprines, this bacterium also infects camels (Abbas and Agab, 2002; Gwida et al., 2012; Sprague et al., 2012; Wernery, 2014). Arabian camels (*Camelus dromedarius*) occur throughout the deserts of North Africa and across the Middle East to North India, a region where they are critical for meat, milk, leather, wool and transport (Wilson, 1984). Camel brucellosis was first reported in 1931 and has since been found in all camel-keeping countries in this region but are particularly well documented for infected herds from Africa and the Arabian Peninsula (Gwida et al., 2012).

Camels are not a primary host for *Brucella* spp. but infections with *B. melitensis* occur due to the co-mingling of camels and ruminant livestock (Sprague et al., 2012). In fact, among the highest prevalence rates in camels have been documented when camel herds are intermixed with ruminants (Musa et al., 2008). Despite this cross-species transmission, epidemiological links between brucellosis in camels and other livestock are poorly understood. Prevalence rates of brucellosis in camels vary widely based on several factors, especially animal husbandry practices (Gwida et al., 2012). Camels can also be infected with *B. abortus*, likely due to the commingling of camel herds with infected cattle (Sprague et al., 2012). Thus, brucellosis prevalence in camels is complex and the role of infections in the primary caprine and bovine hosts must be considered. The pathology of brucellosis infection in camels is poorly known as well. Consistent with findings from other livestock, the bacteria appear to localize in reproductive tissues, lymph nodes, and spleen, causing inflammation, edema, and necrosis (Wernery, 2014). Infection of pregnant camels can result in placental and fetal pathologies resulting in abortion (Narnaware et al., 2017). As with brucellosis in other animals, these abortion events likely disseminate the bacteria broadly and allow for transmission to other livestock and to animal handlers. Not surprisingly, the disease is prevalent in Bedouin in Oman (Scrimgeour et al., 1999).

Several serological tests, such as Rose Bengal, tube and serum agglutination tests and ELISAs that have been optimized for testing cattle are used to determine *Brucella* seroprevalence in camels (Gwida et al., 2012), but epidemiological investigations often stop at this point. Furthermore, the lack of validated serological tests that detect *Brucella* infection in camels pose a challenge to definitive diagnosis. A combination of real-time PCR and serological tests provides a solution to many of the diagnostic challenges (Gwida et al., 2011). To determine the causative species, bacterial culturing from milk, blood or tissues of infected animals is performed to recover bacterial isolates. For *B. melitensis*, subsequent testing is required to distinguish the three biovars that are traditionally assessed in characterizing this species.

Brucellosis is a public health concern throughout the Greater Middle East (Pappas and Memish, 2007) and has been considered “hyperendemic” in Saudi Arabia with ~8,000 reported cases per annum (Memish and Mah, 2001). Comprehensive reporting of the disease throughout the region has been elusive due to limited public health infrastructure in many countries. Refai (2002) documented that brucellosis was ubiquitous throughout the Near East, with highest human incidence in Saudi Arabia, Iran, Syria, Jordan, and Oman. In fact, Western Asia contains among the highest incidence of brucellosis globally (Pappas et al., 2006). Human exposure to brucellosis from camels occurs primarily from contaminated milk (Shimol et al., 2012; Garcell et al., 2016). More broadly across other animal hosts, brucellosis infections most often occur in people working in close contact with animal tissues such as slaughterhouse workers, veterinarians, and farmers (Kaufmann et al., 1980; Whatmore, 2009). Determining the genetic diversity of *B. melitensis* in camels would provide valuable information about disease dispersal and transmission among camels, goats, and sheep in endemic areas, and particularly help better understand the role of camels in human infections.

As part of a brucellosis control program, we collected animal samples from routine surveillance activities. Tissues or bodily fluids were collected and isolates of *B. melitensis* were recovered from camels, goats, sheep, and cattle in the Dhofar governorate of Oman, an area with the highest brucellosis prevalence in the country (El Tahir and Nair, 2011). In 1996, a brucellosis control program that implemented both vaccination and culling of infected animals was initiated in the Dhofar region. As a part of an animal disease surveillance system, the regional Brucellosis Diagnostic Lab in Salalah, Dhofar identified all *Brucella* species that were involved in animal brucellosis cases. Because *B. melitensis* isolates from this region are not well characterized, we took two approaches to assess genetic relationships. First, we placed these isolates into a global phylogeny using single nucleotide polymorphism (SNP)-based genotyping assays specific to major evolutionary lineages of *B. melitensis*. We then genotyped a subset of these samples using multilocus variable number tandem repeats analysis (MLVA), following Huynh et al. (2008), to confirm their placement into well characterized clades. Our results suggest the genetic lineage of *B. melitensis* isolates in camels in Oman extends from North Africa into the Arabian Peninsula.

## Materials and methods

Aborted fetuses, placental membranes and vaginal swabs/discharge from aborted animals, and milk secretions and blood from suspected animal brucellosis cases were collected in the Dhofar governorate of southwestern Oman. Samples were handled under BSL-3 containment at the Animal Health Research Center, Ministry of Agriculture and Fisheries, Sultanate of Oman. Putative *Brucella* samples were inoculated on sheep blood agar plates together with *Brucella* selective supplement (SR 0083; Oxoid, Hampshire, UK) and 2.5% glucose. The plates were incubated at 37°C with (10%) or without CO_2_ for 7 days. Colonies were presumptively identified as *Brucella* by morphology and Gram staining and further biotyped using standard microbiological lab procedures (Alton et al., 1988; OIE, 2012). *Brucella* genus and species identification was confirmed by PCR (Hinic et al., 2008). Thirty-four isolates of *B. melitensis* were recovered from four animal species: camels (*n* = 15), goats (*n* = 8), cattle (*n* = 7), and sheep (*n* = 4) from 1997 to 2010 (Table 1). These samples are stored in the *Brucella* repository of the Animal Health Research Center. Twenty-hour individual broth cultures (~2 × 10^9^/ml) were pelleted by centrifugation (7,500 rpm) for 10 min and genomic DNA was extracted and purified using Qiagen DNeasy Blood and Tissue Kits (Hilden, Germany) following the manufacturer's protocol for Gram-negative bacteria.

**Table 1:** Characteristics of 27 *Brucella melitensis* isolates collected from livestock in Dhofar governorate, Oman.

**Year**	**Collection source**	**Animal**	**Location**	**Catalase**	**Oxidase**	**Urease**	**H_2_S prod**.	**CO_2_ req**.	**Growth on dyes**		**Phage lysis**		**Agglutination with monospecific antisera**	
									**Basic fuchsin**	**Thionin**	**Iz_1_**	**Wb**	**A**	**M**
1997	placental membrane	camel	Salalah	+	+	+	−	−	+	−	+	−	−	+
1999	aborted fetus	camel	Salalah	+	+	+	−	−	+	−	+	−	−	+
1999	milk	camel	Taqah	+	+	+	−	−	+	−	+	−	−	+
1999	milk	camel	Taqah	+	+	+	−	−	+	−	+	−	−	+
2003	fetus	camel	Mirbat	+	+	+	−	−	+	−	+	−	−	+
2004	vaginal swab/fetus	camel	Salalah	+	+	+	−	−	+	−	+	+	−	+
2004	milk/vaginal swab	camel	Salalah	+	+	+	−	−	+	−	+	+	−	+
2004	aborted material	camel	Salalah	+	+	+	−	−	+	−	+	+	−	+
2004	aborted membranes	camel	Mirbat	+	+	+	−	−	+	−	+	−	−	+
2004	fetal stomach	camel	Rakhyut	+	+	+	−	−	+	−	+	+	−	+
2004	fetal stomach	camel	Dalkut	+	+	+	−	−	+	−	+	−	−	+
2004	fetus	camel	Rakhyut	+	+	+	−	−	+	−	+	+	−	+
2005	milk	camel	Thumrayt	+	+	+	−	−	+	−	+	−	−	+
2005	milk	camel	Thumrayt	+	+	+	−	−	+	−	+	−	−	+
2007	stomach content fetus	camel	Muqshin	+	+	+	−	−	+	−	+	−	−	+
1997	fetus (stomach/lungs)	sheep	Mirbat	+	+	+	−	−	+	−	+	−	−	+
1997	fetus	cow	Taqah	+	+	+	−	−	+	−	+	−	−	+
1997	fetus	sheep	Taqah	+	+	+	−	−	+	−	+	−	−	+
1997	fetus	sheep	Taqah	+	+	+	−	−	+	−	+	−	−	+
1997	vaginal discharge	cow	Salalah	+	+	+	−	−	+	−	+	−	−	+
1997	fetus	sheep	Mirbat	+	+	+	−	−	+	−	+	−	−	+
1998	milk /vaginal discharge	cow	Unknown	+	+	+	−	−	+	−	+	−	−	+
1998	milk	cow	Unknown	+	+	+	−	−	+	−	+	−	−	+
1999	fetus	goat	Shalim	+	+	+	−	−	+	−	+	−	−	+
2000	fetus	cow	Unknown	+	+	+	−	−	+	−	+	−	−	+
2000	vaginal discharge	cow	Unknown	+	+	+	−	−	+	−	+	−	−	+
2000	vaginal discharge	cow	Unknown	+	+	+	−	−	+	−	+	−	−	+

*Biotyping indicated that all 27 samples were biovar 1. Seven additional B. melitensis isolates from blood samples from goats were also collected from Saham in the Al Batinah North Governorate in 2010, but were not biotyped*.

A sub-set of MLVA profiles derived from geographically diverse *B. melitensis* isolates causing human infection was generated at the U.S. Centers for Disease Control and Prevention (CDC) and used in the analysis to understand the distribution of *B. melitensis* genotypes in the region. Samples originating from Egypt with the naming designation starting with “E” were collected from an acute febrile illness surveillance study in Egypt as described by Afifi et al. (2005) and a MLVA study of this sub-set of isolates is described by Tiller et al. (2009). The remainder of the patient samples came from reference diagnostic specimens or were recovered from outbreak or support testing by the CDC. Samples were de-identified prior to analyses. Isolation and DNA extraction methods followed those detailed in Tiller et al. (2009). Three reference strains (16 M, 63/9, Rev-1) came from the CDC strain collection but their complete culture history is not known. These strains were MLVA genotyped using the actual isolates rather than using the genome sequence. All strains were minimally passaged after collection from human or animal sources. All culture and manipulation of *Brucella* isolates were conducted in BSL-3 conditions.

**Figure 1:**
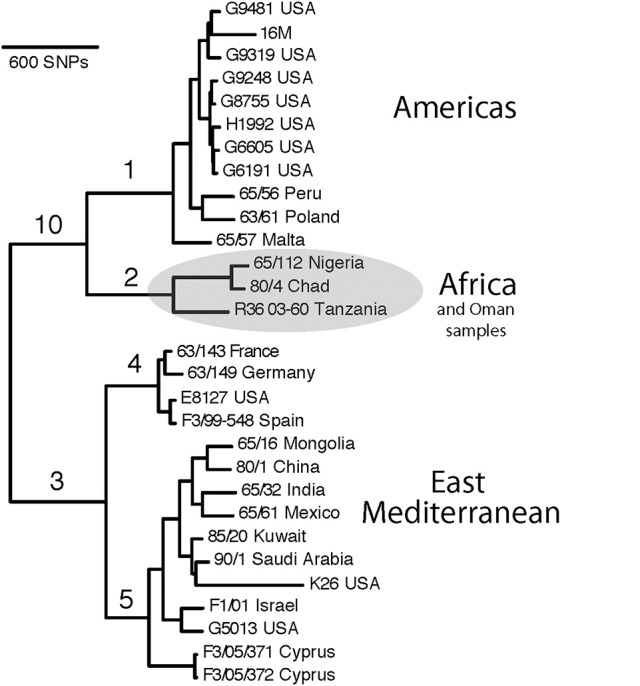
SNP-based phylogenetic tree of *Brucella melitensis* using 29 genomes available at time of assay development. Numbered branches correspond to SNP-based assays for each of the corresponding lineages. Note that the *B. melitensis* lineage known as the W. Mediterranean group (includes the Ether strain), is a basal group and is not shown in this tree. Country names indicate where the isolate was identified, and due to travel and sharing of strains among labs, this is not necessarily the country of origin.

To develop a *B. melitensis* SNP genotyping assay, we conducted an *in silico* analysis of 29 *B. melitensis* genomes that were then available in GenBank and generated a phylogenetic tree using Northern Arizona SNP Pipeline (NASP) (Sahl et al., 2016; Figure 1). We used default parameters for SNP calling for each genome, which included a minimum of 10X coverage at a locus, at least 90% consensus for a SNP allele, and the requirement that the SNP locus was present in all genomes (i.e., no missing data).

Using these genome sequences, flanking regions were aligned using Sequencher 5.0, and Melt-MAMA (Mismatch Amplification Mutation Assays) were designed (Birdsell et al., 2012) targeting at least two randomly selected SNPs on each major branch (Table 2). Changes to primer base composition are detectable in melt-curve analysis, allowing differential fluorescence to determine the allele. Using DNA from the 34 isolates from Omani livestock, we performed Real-Time PCR with SYBR Green incorporation on an Applied Biosystems 7900HT Fast Real-Time PCR system. We ran 5 μL reactions with 1 μL DNA standardized to 2 ng/μL, when possible, and 4 μL of PCR reagents. Final concentration per reaction was 1X ABI SYBR Green Universal PCR Master Mix, and 0.15 μM each of the derived MAMA primer, ancestral MAMA primer, and reverse consensus primer. In practice this converts to 1.28 μL molecular grade water, 2.5 μL of 2X Universal PCR Master mix, and 0.08 μL each of the three primers at 10 μM each. In melt-MAMA reactions, the derived or ancestral primer containing the SNP allele more effectively amplifies in a competing reaction. Thermo-cycling conditions were as follows: initial UNG activation of 2 min at 50°C, a hot start of 10 min at 95°C, followed by 40 PCR cycles of 15 s at 95°C for denaturation and 1 min at 60°C for annealing, and a final stage of 15 s at 95°C, 15 s at 60°C, and 15 s at 95°C. Alleles were readily distinguished by the melting step and were confirmed with positive controls for each allele state (Birdsell et al., 2012). The SNP genotyping assays were run on all 34 Omani livestock *B. melitensis* samples.

**Table 2:** Assay design parameters for primer sets targeting 6 major branches of *Brucella melitensis*.

**Branch**	**Reference genome position**	**SNP state**	**Primer**	**5′–>3′**	**State**	**% GC content**	**Primer Tm°C**	**Primer length (bp)**	**Amplicon length (bp)**	**Amplicon Tm°C**
1	6028	A/G	F1	ggggcggggcggggcCCGGCGAAATGCTGGCGaTa	D	60	56	20	42	74
			F2	CCGGCGAAATGCTGGCGtTg	A	65	58	20		
			C	GATGCGTATAGCCTTCCTCGC	C	57	56	21		
2	1169400	A/G	F1	ggggcggggcggggcGCAGAAGCGCACTGGAATATgTa	D	48	55	23	58	72
			F2	GCAGAAGCGCACTGGAATATaTg	A	48	55	23		
			C	GGTTAAAATATGCTGTGCTGTACAGGG	C	44	58	27		
3	1127740	C/T	F1	ggggcggggcggggcCGTAACAGGCAGCAATCTGCAgTc	D	54	59	24	50	73
			F2	CGTAACAGGCAGCAATCTGCAcTt	A	50	57	24		
			C	TCAAACTATTAAGGGGTCGTTCCGG	C	55	54	25		
4	870030	T/C	F1	ggggcggggcggggcCGCGGGTTTCTTCATCCAGAAtGt	D	63	55	24	51	75
			F2	CGCGGGTTTCTTCATCCAGAAgGc	A	58	61	24		
			C	GCCGGGGCGACATCATAGATCG	C	64	60	22		
5	842276	T/C	F1	ggggcggggcggggcGCGCCTCCTGCTGCCTcCt	D	74	60	19	49	72
			F2	GCGCCTCCTGCTGCCTaCc	A	74	59	29		
			C	GAATCATTATCGTTCAGATACATAAAGCC	C	34	56	29		
10	747768	A/C	F1	ggggcggggcggggcGGCGCGGAGCCATATTGgAa	D	60	56	20	55	73
			F2	GGCGCGGAGCCATATTGcAc	A	65	58	20		
			C	CCTTTAACCCTAGCAATTGGAGGAAC	C	46	58	26		

*Reference genome is 16 M (NCBI RefSeq accession numbers NC_003317.1 and NC_003318.1). Branch numbers correspond to branch labels in Figure 1. State column refers to Derived (D), Ancestral (A), and Consensus (C), state of the SNP targeted by that specific primer. Tm is the melting temperature for the primers or the amplicon*.

We also performed MLVA on the 15 camel isolates following the methods of Huynh et al. (2008). Briefly, this is a 15 locus VNTR panel that uses four multiplex PCRs with fluorescently labeled forward primers (6-FAM, NED, PET, or VIC) and unlabeled reverse primers. Fragment sizes for each locus were visualized by capillary electrophoresis on an ABI 3130 Genetic Analyzer and converted to a repeat number corresponding to each fragment size using the LIZ 1200 size standard in GeneMapper version 4.0. As detailed by Tiller et al. (2009), the MLVA approach we used compares favorably to the more widely used MLVA approach (Le Fleche et al., 2006), although the latter has an expansive database of MLVA genotypes (http://mlva.u-psud.fr/brucella/).

## Results

In this work, we used SNPs discovered in whole genome comparisons to develop multiple SNP assays that distinguish six major branches in *B. melitensis*. We present details on the assays that provided the greatest peak separation for SNPs found on each branch (Table 2). Of particular interest to this study was the phylogenetic placement of isolates from Omani livestock; all 34 of these Omani isolates are part of the clade on the assay branch 2 (Figure 1). The finding that Omani livestock samples all came from a distinct branch in the *B. melitensis* phylogeny indicates that these animals all contain relatively closely related isolates from a single lineage.

SNP analyses indicate that all of our *B. melitensis* isolates from Omani camels belong to a distinct clade that also contains isolates originating in Africa (Nigeria, Chad, Tanzania). This key finding was unexpected, as one would predict that isolates from the Arabian Peninsula would be part of the E. Mediterranean lineage that predominates the region (Gyuranecz et al., 2016). Thus, SNP analysis indicates that Omani livestock isolates are part of a group of *B. melitensis* that is related to isolates from Africa and distantly related to most other isolates from the Middle East. Moreover, it suggests that the lineages of *B. melitensis* in Egypt and probably throughout much of N. Africa are distinct from most strains from the rest of the Middle East. Our SNP assays also identify two major groups within the E. Mediterranean clade, branches 4 and 5, suggesting substructure within this group. Interestingly, the African clade isolates are more closely related to isolates from the Americas clade than the E. Mediterranean clade in this rooted tree, a pattern not seen in MLVA (Gyuranecz et al., 2016), likely due to the higher resolution of whole genome sequencing.

MLVA results were compared to our database of MLVA genotypes from the Middle East (Huynh et al., 2008; Tiller et al., 2009). We utilized all MLVA genotypes from the African clade and a representative sample of genotypes from the E. Mediterranean and Americas clades (Table 3). A fourth lineage, W. Mediterranean, is not part of our collection, nor was it detected in our sampling so was not included in our phylogeny. The W. Mediterranean clade is largely limited to Italy (Garofolo et al., 2013) but does occur in other countries, including along the Mediterranean (Lounes et al., 2014). Low DNA quality prevented us from running MLVA on the 12 other animal isolates from Oman but this DNA was still of sufficient quality to SNP-based analysis on Real-Time PCR due to the smaller amplicons sizes and higher sensitivity of these Real-Time assays (Birdsell et al., 2012). The 10 most stable of the 15 Variable Number Tandem Repeat (VNTR) loci were used in our analyses (VNTRs: 1, 3, 7, 14, 16, 20, 21, 25 27, 28). Limiting VNTR loci to the most stable markers, or placing greater weighting on these markers, is a common practice in *Brucella* MLVA (e.g., Al Dahouk et al., 2007a). Such a practice reduces homoplasy and allows for understanding deeper phylogenetic relationships but potentially sacrifices more recent epidemiological connections (Keim et al., 2004); this potential loss of resolution was not a concern for our study because we were focused on these deeper connections.

**Table 3:** MLVA-10 genotypes for 221 isolates of *Brucella melitensis* from the Middle East. Isolates from additional regions added for context.

**Sample ID**	**Country**	**Host**	**Lineage**	**VNTR locus**									
				**21**	**14**	**28**	**1**	**16**	**7**	**3**	**27**	**20**	**25**
1652	Afghanistan	goat	E. Med.	100	123	191	267	225	320	322	389	453	496
1657	Afghanistan	sheep	E. Med.	100	123	215	219	225	308	322	413	453	496
1761	Afghanistan	sheep	E. Med.	100	123	191	267	225	320	322	389	453	496
2011756220	Afghanistan	human	E. Med.	100	123	191	202	225	320	322	381	453	496
2011756221	Afghanistan	goat	E. Med.	100	123	191	219	225	320	322	381	453	496
2013004349	Afghanistan	human	E. Med.	100	123	191	235	225	320	322	381	453	496
25	Afghanistan	human	Americas	100	123	174	227	258	327	370	373	453	496
27	Afghanistan	human	Americas	100	123	174	227	258	327	370	373	453	496
29	Afghanistan	human	Americas	100	123	174	227	258	327	370	373	453	496
31	Afghanistan	human	Americas	100	123	174	227	258	327	370	373	453	496
2008010924	Bosnia	human	E. Med.	100	123	208	227	225	320	386	438	453	496
2011756294	Bosnia	human	E. Med.	100	123	232	251	225	320	354	413	453	496
2008018505	Egypt	human	Africa	100	123	215	219	250	320	403	413	441	496
E1-ABS-9258	Egypt	human	Africa	100	123	215	300	250	316	370	397	441	496
E10-ALX-0769	Egypt	human	Africa	100	123	184	194	250	316	386	446	441	496
E11-ASW-0309	Egypt	human	Africa	100	123	215	227	250	316	419	389	441	496
E12-ABS-9517	Egypt	human	Africa	100	123	191	219	250	316	394	430	441	496
E13-FAY-9671	Egypt	human	Africa	100	123	223	235	250	316	394	397	441	496
E14-BEN-0044	Egypt	human	Africa	100	123	208	314	250	316	394	397	441	496
E15-SHB-10112	Egypt	human	Africa	100	123	174	235	250	316	419	430	441	496
E16-ZAG-0133	Egypt	human	Africa	100	123	215	243	250	316	419	405	441	496
E17-PRS-0241	Egypt	human	Africa	100	123	199	235	250	316	460	413	441	496
E18-SHB-1482	Egypt	human	Africa	100	123	215	243	250	316	403	430	441	496
E19-ALX-0996	Egypt	human	Africa	100	123	199	219	250	316	444	413	441	496
E2-MAL-9273	Egypt	human	Africa	100	123	208	219	250	316	411	430	441	496
E20-MAL-0941	Egypt	human	Africa	100	123	215	227	250	316	435	405	441	496
E21-ABS-10208	Egypt	human	Africa	100	123	208	211	250	316	330	381	441	496
E22-QEN-0166	Egypt	human	Africa	100	123	223	267	250	316	386	397	441	496
E23-ABS-10654	Egypt	human	Africa	100	123	199	243	250	316	338	381	441	496
E24-ALX-1734	Egypt	human	Africa	100	123	199	243	250	316	354	405	441	496
E25-MAL-1462	Egypt	human	Africa	100	123	191	235	250	316	419	413	441	496
E26-SHB-2267	Egypt	human	Africa	100	123	208	243	250	316	378	430	441	496
E27-ALX-2000	Egypt	human	E. Med.	95	123	215	219	217	316	322	413	393	496
E28-SHB-2340	Egypt	human	Africa	95	123	191	227	250	316	322	405	393	496
E29-ASW-1381	Egypt	human	Africa	100	123	199	211	250	316	403	381	441	496
E3-SHB-0395	Egypt	human	Africa	100	123	199	219	250	316	346	405	441	496
E30-AST-0977	Egypt	human	Africa	100	123	199	267	258	316	394	397	441	496
E31-AST-1030	Egypt	human	E. Med.	95	123	215	243	217	316	322	381	425	496
E32-QEN-0364	Egypt	human	Africa	100	123	215	227	250	316	403	373	441	496
E33-ALX-2307	Egypt	human	Africa	100	123	199	267	250	316	419	389	441	496
E34-MAL-1821	Egypt	human	Africa	100	123	191	251	250	316	370	413	441	496
E35-SHB-2580	Egypt	human	Africa	100	123	208	235	250	316	452	430	441	496
E36-ALX-2198	Egypt	human	Africa	100	123	191	243	250	316	435	397	441	496
E37-QEN-0388	Egypt	human	Africa	100	123	223	243	250	316	411	373	441	496
E38-ASW-1553	Egypt	human	Africa	100	123	199	251	250	316	394	389	441	496
E39-ALX-0077	Egypt	human	Africa	100	123	208	283	250	316	428	389	441	496
E4-QEN-0062	Egypt	human	Africa	100	123	223	219	250	316	386	373	441	496
E40-ABS-9024	Egypt	human	Africa	100	123	208	211	250	316	419	389	441	496
E41-SOH-9085	Egypt	human	Africa	100	123	248	243	250	316	346	381	441	496
E42-SOH-0002	Egypt	human	Africa	100	123	248	243	250	316	346	381	441	496
E43-SHB-9203	Egypt	human	Africa	100	123	199	227	250	316	419	397	441	496
E44-SHB-9204	Egypt	human	Africa	100	123	208	211	258	316	419	397	441	496
E45-SHB-0492	Egypt	human	Africa	100	123	191	227	250	316	468	405	441	496
E46-SHB-0496	Egypt	human	Africa	100	123	191	267	250	316	403	430	441	496
E47-ZAG-0127	Egypt	human	Africa	100	123	199	235	258	316	444	397	441	496
E48-MAL-0966	Egypt	human	Africa	100	123	208	219	250	316	403	397	441	496
E49-MAL-0958	Egypt	human	Africa	100	123	215	243	250	316	419	397	441	496
E5-ABS-9281	Egypt	human	Africa	100	123	191	283	250	316	419	446	441	496
E50-SHB-1580	Egypt	human	Africa	100	123	199	227	250	316	346	430	441	496
E51-SHB-1572	Egypt	human	Africa	100	123	191	243	250	316	354	430	356	496
E52-FAY-10244	Egypt	human	Africa	100	123	191	291	258	316	370	389	441	496
E53-FAY-10257	Egypt	human	Africa	100	123	191	219	258	316	394	389	441	496
E54-BEN-0182	Egypt	human	Africa	100	123	191	227	250	316	452	381	441	496
E55-MAL-1111	Egypt	human	Africa	100	123	215	227	250	316	444	381	441	496
E56-SHB-2081	Egypt	human	Africa	100	123	191	194	250	316	394	389	441	496
E57-ASW-10776	Egypt	human	Africa	100	123	208	202	250	316	411	389	441	496
E58-SHB-2177	Egypt	human	Africa	100	123	199	259	250	316	394	397	441	496
E59-ASW-1220	Egypt	human	Africa	100	123	208	259	250	316	386	405	441	496
E6-SHB-0407	Egypt	human	Africa	100	123	199	267	250	316	403	405	441	496
E60-ASW-1237	Egypt	human	Africa	100	123	199	211	250	316	403	381	441	496
E61-AST-1053	Egypt	human	Africa	100	123	208	235	258	316	394	397	441	496
E62-AST-1008	Egypt	human	Africa	100	123	208	194	258	316	394	397	441	496
E63-AST-1085	Egypt	human	Africa	100	123	199	243	258	316	403	397	441	496
E64-ALX-4	Egypt	human	Africa	100	123	191	202	250	316	403	397	441	496
E65-ALX-107	Egypt	human	Africa	100	123	215	235	250	316	435	389	441	496
E66-MAL-104	Egypt	human	Africa	100	123	199	259	250	316	468	397	441	496
E67-MAL-135	Egypt	human	Africa	100	123	208	243	250	316	460	381	441	496
E68-ABS-52	Egypt	human	Africa	100	123	208	235	258	316	362	389	441	496
E69-ABS-157	Egypt	human	Africa	100	123	199	235	250	316	428	413	441	496
E7-ALX-0404	Egypt	human	Africa	100	123	208	283	250	316	378	397	441	496
E70-ABS-49	Egypt	human	Africa	100	123	199	251	250	316	394	446	441	496
E71-AFI-ABS-134	Egypt	human	Africa	100	123	215	211	217	316	452	413	441	496
E72-MAL-305	Egypt	human	Africa	100	123	208	219	250	316	378	430	441	496
E73-ALX-458	Egypt	human	Africa	100	123	199	259	250	316	386	389	441	496
E74-MAL-293	Egypt	human	Africa	100	123	208	276	250	316	370	413	441	496
E75-ABS-93	Egypt	human	Africa	100	123	199	235	250	316	444	413	441	496
E76-ABS-211	Egypt	human	Africa	100	123	199	235	258	316	403	389	405	496
E77-ALX-467	Egypt	human	Africa	100	123	199	235	250	316	370	389	441	496
E78-ALX-468	Egypt	human	Africa	100	123	208	211	258	316	386	397	441	496
E79-MAL-335	Egypt	human	Africa	100	123	215	211	250	316	386	413	441	496
E8-SHB-0676	Egypt	human	Africa	100	123	191	259	250	316	419	389	441	496
E80-MAL-353	Egypt	human	Africa	100	123	199	219	250	316	394	389	441	496
E81-ALX-535	Egypt	human	Africa	100	123	199	259	250	316	394	389	441	496
E82-ALX-532	Egypt	human	Africa	100	123	191	211	258	316	411	381	441	496
E83-ASW-84	Egypt	human	Africa	100	123	199	251	250	316	378	381	441	496
E9-FAY-9445	Egypt	human	Africa	100	123	199	267	250	316	419	389	441	496
3000409149	Ethiopia	human	Americas	100	123	215	194	258	327	362	381	453	496
2010022407	Greece	human	E. Med.	100	123	215	235	225	320	370	405	453	498
2006012884	Iran	human	E. Med.	100	123	208	243	225	320	322	397	453	496
2006012885	Iran	human	E. Med.	100	123	208	243	225	320	322	397	453	496
2010022413	Iran	human	Americas	100	123	174	219	258	320	362	405	453	496
3000027166	Iran	human	E. Med.	100	123	199	227	225	320	322	413	453	496
2004017806	Iraq	human	E. Med.	100	123	191	219	225	320	322	413	453	496
2008724248	Iraq	human	E. Med.	100	123	215	211	225	320	338	430	453	496
2012005317	Iraq	human	E. Med.	100	123	191	251	225	320	362	381	453	496
2012017239	Iraq	human	Intermediate	100	123	184	211	225	320	386	389	453	496
2013746956	Iraq	human	E. Med.	100	123	208	219	225	320	354	389	453	496
2013833054	Iraq	human	Intermediate	100	123	199	211	225	320	346	389	453	496
2014001382	Iraq	human	Intermediate	100	123	199	194	225	320	386	397	453	496
2014001698-2	Iraq	human	Intermediate	100	123	199	194	225	320	386	397	453	496
2014001698-3	Iraq	human	Intermediate	100	123	199	194	225	320	386	397	453	496
1988035349	Israel	human	Africa	100	123	208	211	225	320	378	389	441	496
1988035350	Israel	human	E. Med.	100	123	191	235	225	320	362	405	453	496
1988035351	Israel	human	E. Med.	100	123	199	219	225	320	386	389	453	496
1988035352	Israel	human	E. Med.	100	123	199	219	225	320	386	381	453	496
1988035353	Israel	human	E. Med.	100	123	199	219	225	320	386	389	453	496
1988035354	Israel	human	Africa	100	123	208	202	225	320	378	389	441	496
1988035355	Israel	human	E. Med.	100	123	191	235	225	320	362	405	453	496
1988035356	Israel	human	E. Med.	100	123	191	235	225	320	362	405	453	496
1988035357	Israel	human	E. Med.	100	123	191	235	225	320	362	405	453	496
1988035358	Israel	human	E. Med.	100	123	199	219	225	320	386	389	453	496
1988035359	Israel	human	E. Med.	100	123	191	235	225	320	362	405	453	496
1988035360	Israel	human	Intermediate	100	123	199	211	225	320	386	389	453	496
1988035361	Israel	human	Africa	100	123	199	211	225	320	378	389	453	496
1988035362	Israel	human	Intermediate	100	123	199	211	225	320	386	389	453	496
2014002005	Israel	human	E. Med.	100	123	208	227	225	320	322	389	453	496
2002018756	Italy	human	Africa	100	123	199	243	250	320	394	405	441	496
2003018302	Italy	human	Africa	100	123	199	243	250	320	394	405	441	496
3000015492	Jordan	human	E. Med.	100	123	223	202	225	320	362	373	453	496
3000015245	Kazakhstan	human	E. Med.	100	123	199	251	225	320	322	405	453	496
2012016719	Lebanon	human	E. Med.	100	123	191	276	225	320	378	389	453	496
L1	Libya	human	Africa	100	123	199	267	258	316	394	397	441	496
2011756293	Mexico	human	Americas	100	123	174	202	250	320	362	381	453	496
2012005445	Mexico	human	Americas	100	123	174	235	250	327	362	389	453	496
2013005190	Mexico	human	Africa	100	115	184	243	241	320	403	373	441	478
2013005440	Mexico	human	Americas	100	123	174	227	250	327	354	389	453	496
2013007561	Mexico	human	Americas	100	123	174	227	241	327	354	389	453	496
2013746874	Mexico	human	Americas	100	123	174	235	241	327	346	389	453	496
2014001235	Mexico	human	Americas	100	123	174	227	250	320	354	413	453	496
2014001327	Mexico	human	Americas	100	123	174	202	241	327	362	389	453	496
3000015243	Mexico	human	Americas	100	123	174	227	250	327	354	381	453	496
3000015269	Mexico	human	Americas	100	123	174	227	241	327	346	389	453	496
3000023891	Mexico	human	Americas	100	123	174	219	250	327	362	381	453	496
3000023892	Mexico	human	Americas	100	123	174	235	241	327	346	389	453	496
3000023893	Mexico	human	Americas	100	123	174	219	250	327	362	381	453	496
3000023969	Mexico	human	Americas	100	123	174	235	250	327	394	413	453	496
3000026676	Mexico	human	Americas	100	123	174	202	250	327	338	389	453	496
3000050553	Mexico	human	Americas	100	123	174	211	258	327	362	373	453	496
3000050738	Mexico	human	Americas	100	123	174	211	258	327	362	373	453	496
3000404015	Mexico	human	Americas	100	123	174	267	250	327	370	373	453	496
3000404142	Mexico	human	Americas	100	123	174	227	250	327	354	405	453	496
3000407000	Mexico	human	Americas	100	123	174	219	250	327	354	397	453	496
3000496880	Mexico	human	Americas	100	123	174	211	250	327	362	381	453	496
3000496881	Mexico	human	Americas	100	123	174	211	250	327	362	381	453	496
BTRU #1501	Mexico	human	Americas	100	123	174	227	241	327	394	381	453	496
MEX349	Mexico	human	Americas	100	124	175	219	265	325	362	397	453	493
MEX350	Mexico	human	Americas	100	124	175	219	249	325	387	373	453	493
MEX351	Mexico	human	Americas	100	124	175	219	249	325	386	373	453	493
MEX352	Mexico	human	Americas	100	124	175	178	241	325	362	405	453	493
Bruc-VRC-1	Oman	camel	Africa	101	124	191	202	258	318	380	389	440	491
Bruc-VRC-10	Oman	camel	Africa	101	124	199	186	250	318	348	374	440	491
Bruc-VRC-11	Oman	camel	Africa	101	124	199	186	250	316	348	374	440	492
Bruc-VRC-12	Oman	camel	Africa	101	124	199	259	250	316	348	374	440	491
Bruc-VRC-13	Oman	camel	Africa	101	124	207	235	250	318	348	413	440	491
Bruc-VRC-14	Oman	camel	Africa	101	243	199	124	250	317	348	413	440	491
Bruc-VRC-15	Oman	camel	Africa	101	124	191	226	258	316	356	397	440	491
Bruc-VRC-2	Oman	camel	Africa	101	123	191	235	250	318	340	405	440	491
Bruc-VRC-3	Oman	camel	Africa	101	124	191	227	258	318	356	398	440	492
Bruc-VRC-4	Oman	camel	Africa	101	124	191	210	258	318	404	382	440	491
Bruc-VRC-5	Oman	camel	Africa	101	124	199	210	250	301	397	389	440	491
Bruc-VRC-6	Oman	camel	Africa	101	124	207	227	258	318	356	390	440	491
Bruc-VRC-7	Oman	camel	Africa	101	124	199	226	258	318	356	389	439	491
Bruc-VRC-8	Oman	camel	Africa	101	124	207	227	258	317	356	389	439	491
Bruc-VRC-9	Oman	camel	Africa	101	124	207	227	258	318	356	390	439	492
2010023909	Qatar	human	E. Med.	100	123	191	194	225	320	370	405	453	496
2010023910	Qatar	human	E. Med.	100	123	199	227	225	320	322	405	464	496
2013002770	Qatar	human	Africa	100	123	215	211	250	320	354	397	441	496
Q1-AT-BR-001	Qatar	human	Africa	100	123	215	202	250	316	378	405	441	496
Q10-AT-BR-010	Qatar	human	Africa	100	123	208	211	233	316	322	397	464	496
Q11-AT-BR-011	Qatar	human	E. Med.	100	123	191	227	233	316	346	413	453	496
Q12-AT-BR-012	Qatar	human	E. Med.	100	123	208	219	233	316	322	389	453	496
Q13-AT-BR-013	Qatar	human	Africa	100	123	215	251	250	316	419	389	441	496
Q14-AT-BR-014	Qatar	human	E. Med.	100	123	191	227	233	316	378	389	453	496
Q15-AT-BR-015	Qatar	human	Africa	100	123	199	235	233	316	370	397	453	496
Q16-AT-BR-016	Qatar	human	E. Med.	100	123	191	227	233	316	322	389	464	496
Q17-AT-BR-017	Qatar	human	Africa	100	123	184	267	233	316	378	381	441	496
Q2-AT-BR-002	Qatar	human	Africa	100	123	199	235	233	316	338	397	453	496
Q3-AT-BR-003	Qatar	human	Africa	100	123	215	202	217	316	370	405	441	496
Q4-AT-BR-004	Qatar	human	E. Med.	100	123	191	194	233	316	346	413	453	496
Q5-AT-BR-005	Qatar	human	Africa	100	123	215	202	217	316	370	405	441	496
Q6-AT-BR-006	Qatar	human	Africa	100	123	199	235	258	316	378	397	441	496
Q7-AT-BR-007	Qatar	human	Africa	100	123	232	186	258	316	444	457	441	496
Q8-AT-BR-008	Qatar	human	E. Med.	100	123	199	227	233	316	322	405	453	496
Q9-AT-BR-009	Qatar	human	E. Med.	100	123	191	235	233	316	322	389	464	496
2011756247	reference	type strain 16 M	Americas	100	123	174	227	233	327	346	373	453	496
2011756235	reference	type strain 63/9	E. Med.	100	123	215	267	225	308	322	389	453	496
2010022409	reference	Rev-1 strain	Americas	100	123	174	227	258	327	370	381	453	496
2011019381	Saudi Arabia	human	E. Med.	100	123	232	276	225	320	322	413	464	496
2011756376	Saudi Arabia	human	E. Med.	100	123	208	235	225	320	322	405	464	496
2013008314	Saudi Arabia	human	E. Med.	100	123	199	243	225	320	322	397	453	496
2013008432	Saudi Arabia	human	E. Med.	100	123	199	227	225	320	322	381	453	496
2013012794	Saudi Arabia	human	E. Med.	100	123	215	235	225	320	322	422	464	496
2013016252	Saudi Arabia	human	E. Med.	100	123	199	227	225	320	322	381	453	496
2014003496	Saudi Arabia	human	E. Med.	100	123	223	227	225	320	322	413	464	496
3000015437	Saudi Arabia	human	E. Med.	100	123	191	219	225	320	322	389	453	496
3000015487	Saudi Arabia	human	Americas	100	123	199	227	225	327	386	389	453	496
3000015489	Saudi Arabia	human	E. Med.	100	123	208	235	225	320	322	389	453	496
F4018	Saudi Arabia	human	E. Med.	100	124	207	219	225	317	322	389	452	492
3000358781	Senegal	human	Americas	100	123	174	202	241	320	330	381	453	496
2011756228	Somalia	human	E. Med.	95	123	191	243	225	327	338	389	453	496
2013746792	Somalia	human	E. Med.	95	123	191	243	225	327	338	389	453	496
2013746793	Somalia	human	E. Med.	95	123	191	243	217	327	362	381	453	496
2004017844	Syria	human	Africa	100	123	184	211	225	324	338	397	441	498
2004017845	Syria	human	E. Med.	100	123	199	227	225	320	370	405	441	496
2008724251	Syria	human	Americas	100	123	174	227	250	327	370	381	453	496
2010724533	Syria	human	E. Med.	100	123	208	235	225	320	362	397	453	496
2013746965	Syria	human	Americas	100	123	174	227	258	327	370	373	453	496
2006006287	Turkey	human	Africa	100	123	184	211	233	316	386	430	441	496
2009027624	Turkey	human	E. Med.	100	123	191	202	225	320	346	397	453	496
2013008341	UAE	human	E. Med.	100	123	199	243	225	320	354	413	356	496
3000015259	Uzbekistan	human	E. Med.	100	123	199	243	225	320	322	381	453	496

**Figure 2:**
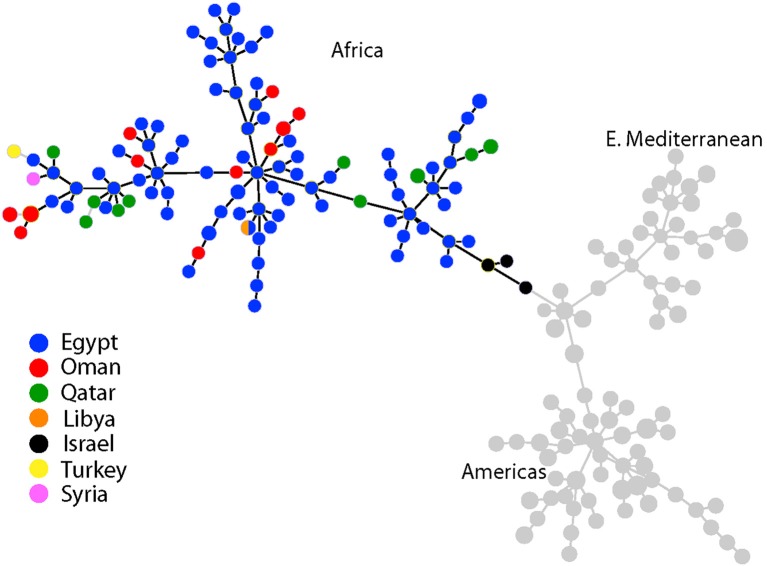
Minimum spanning tree of 221 *Brucella melitensis* isolates based on MLVA-10 genotyping. Country of origin for isolates from the Middle East is represented by the colored circles. Additional isolate details are found in Table 3.

*Country refers to the suspected origin of brucellosis infection although human travel and trade in contaminated animal products may obscure the true origin. Lineage refers to the MLVA branch from Figure 2; seven isolates were classified as Intermediate due to genotypes not assigned to one of the three main lineages we investigated (Africa, Americas, E. Mediterranean). VNTR loci follow the numbering of Huynh et al. (2008). Strains not definitively assigned to a lineage were considered Intermediate*.

Three distinct lineages of *B. melitensis* were found in our sampling, consistent with geographic groupings of Americas, E. Mediterranean, and Africa clades. Al Dahouk et al. (2007a) first identified the Americas and E. Mediterranean clades but used a different MLVA genotyping scheme (Le Fleche et al., 2006). Subsequent work by Gyuranecz et al. (2016), identified another lineage, the Africa clade. This consistency between the two MLVA schemes, as well as support from whole genome analyses, suggests that either scheme is capable of identifying these major lineages although differentiation of the Africa clade is more pronounced with the Huynh et al. (2008) MLVA scheme. VNTR genotypes were compared using minimum spanning trees in Phyloviz 2.0 (Nascimento et al., 2017).

Using MLVA genotyping, the VNTR diversity we observed in these 15 camel isolates divide into 3–6 distinct lineages, depending on clustering criteria (Figure 2). Use of all 15 MLVA loci gave similar overall patterns (data not shown). All camel isolates from Oman, except for one highly similar to a human isolate from Qatar (Q7), are closely related to samples from human patients from Egypt, typically differing from each other by only 1–3 VNTR mutations; indicating that highly related strains circulating across the region in both livestock and humans. Our MLVA results also support the SNP analyses that the Omani camel isolates have greatest similarity to *B. melitensis* isolates from North Africa. Omani camel isolates are related to isolates originating from humans in Egypt, sometimes separated by only one VNTR repeat. Epidemiological trace back may allow for identification of these potential animal to human transmission events. We also note the presence of African lineage isolates in other countries in the region, including Libya, Israel, Syria, Turkey, and Qatar.

## Discussion

Our study highlights the presence of a unique lineage of *B. melitensis* in the Middle East, provides assays to quickly identify the strain types circulating and causing animal and human brucellosis in the region, and suggests that substantial diversity remains to be uncovered in Africa. The SNP assays we designed, particularly those for branch 2, allow for rapid identification of African lineage strains and thus can be used to focus on finding new genetic variation among this poorly sampled group. When samples are identified as part of the African lineage, MLVA can then be utilized for higher resolution analyses, such as understanding the relationships among African isolates and for epidemiological investigations.

### SNP genotyping

The SNP-based results from the whole genome analyses and SNP genotyping support the finding that an African lineage of *B. melitensis* exists in the Arabian Peninsula (e.g., Oman and Qatar), and other nearby countries such as Israel, Turkey, and Syria. Although the African lineage appears to be uncommon elsewhere, it nonetheless has been introduced into many Middle Eastern countries. The two SNP assays specific for branch 2 can quickly identify isolates that are part of this unique African lineage, allowing for a determination of the spread of this clade.

We emphasize that the African clade is a phylogenetic assignment and not a geographic one, and that not all isolates from Africa will be a part of this clade. Accordingly, isolates from Algeria in western North Africa were predominantly from the W. Mediterranean clade (Lounes et al., 2014), suggesting a connection to Europe (especially Italy) rather than the rest of North Africa or other parts of the Middle East. Our SNP findings, and those of Georgi et al. (2017), highlight the power of whole genome analyses and this genomic approach is able to clearly distinguish isolates from the African and E. Mediterranean clades as evolutionarily distinct lineages.

### MLVA genotyping

The MLVA groupings show broad and consistent geographic representations of MLVA types from the Americas, E. Mediterranean clade (consisting primarily of West/Central Asia and Middle Eastern isolates), and Africa. Although this method uses different loci than SNP approaches, these groupings match those from the SNP-based genotyping. Using the MLVA method of Le Fleche et al. (2006), *B. melitensis* isolates from the United Arab Emirates from camels, cattle, and a goat had genotypes from the E. Mediterranean clade but also contained isolates from four camels and a gazelle from the African clade (Gyuranecz et al., 2016). Indeed, the expectation for *B. melitensis* isolates collected from humans or livestock from the Arabian Peninsula and most of the Middle East is membership in the E. Mediterranean clade (Al Dahouk et al., 2007a; Kattar et al., 2008; Kilic et al., 2011). MLVA and whole genome analyses indicate that the E. Mediterranean clade is the principal lineage in the region, continuing from the Middle East along the Mediterranean and into and throughout Asia (Jiang et al., 2011; Tan et al., 2015; Tay et al., 2015). Whole genome comparisons from brucellosis patients infected in the Middle East support this predominance of the E. Mediterranean clade but also identify three isolates of Somali origin from an Africa clade (Georgi et al., 2017). Despite its broad sampling and thousands of genotypes for *B. melitensis*, the MLVA database (http://mlva.u-psud.fr/brucella/) contains few representatives from North Africa so it appears to be currently missing much of this genetic diversity. We encourage researchers using the Le Fleche MLVA scheme to genotype samples from this region for a better global understanding of this lineage, after first using our SNP-based assays to identify this clade.

### Resolving apparent inconsistencies

Our findings challenge common approaches used in *Brucella* epidemiology. While we detail our results from traditional phenotyping approaches, these data are typically not informative for understanding the genetic relationships of isolates within *B. melitensis*. For example, distinguishing the three biovars of *B. melitensis* provides limited resolution when attempting to establish epidemiological links between outbreaks; likely due to the limited association between genotype and phenotype (biovar) in this species (Whatmore et al., 2016). In fact, isolates from biovars 1, 2, and 3 can be closely related genetically (e.g., Jiang et al., 2011) and isolates from the same biovar can be distantly related and from evolutionarily distinct clades (e.g., De Massis et al., 2015). Apparent discrepancies also occur for the country of origin for some isolates; a handful of SNP-based and MLVA genotypes appear to not match the expected clade corresponding to the region. For example, in the SNP phylogeny an isolate from Poland is in the Americas clade, and samples from the USA are in the E. Mediterranean clade. Such discordance is expected. First, as mentioned previously these groupings are phylogenetic assignments and should not be misconstrued as geographic origins. In addition, epidemiological histories of various strains are not always precisely known and the country names may indicate where the isolate was identified and not necessarily the country of origin. Finally, international travel of patients infected with brucellosis or movement of infected animals may potentially obscure the country of origin unless detailed epidemiological data are available (Al Dahouk et al., 2007b; Garofolo et al., 2013; De Massis et al., 2015; Georgi et al., 2017).

### Brucellosis management

Disease management approaches involving vaccination, treatment with antibiotics, and test-and-slaughter programs for camels can lower disease incidence and even eliminate brucellosis from some farms (Radwan et al., 1995; Abbas and Agab, 2002; Wernery, 2014). Nonetheless, comprehensive elimination of *B. melitensis* in camels from larger regions will require brucellosis management in goats and sheep. Despite calls for the elimination of brucellosis from the Arabian Peninsula for over two decades (Tabbara, 1993), the disease persists throughout the region and indeed continues to be a successful pathogen throughout much of world. High prevalence rates in sheep, goats and cattle, and the hundreds of millions of these animals in the region make brucellosis elimination difficult (Refai, 2002). Although the economic cost of brucellosis to livestock production in Oman is not well-known, globally the economic burden on both animal and human health is significant and far reaching (Seleem et al., 2010). Understanding historic and contemporary movement of animals infected with brucellosis is an important step in improving disease management strategies.

### Brucellosis movement

The presence of an African lineage from North Africa in the Arabian Peninsula indicates the interconnectedness of livestock in the Greater Middle East, likely due to historical trade and movement between the two regions, although the E. Mediterranean lineage still predominates. Contemporary introductions of *B. melitensis* also appear to occur—the four camels with the African clade genotypes in the United Arab Emirates came from Sudan (Gyuranecz et al., 2016)—connectedness previously suggested by Wernery (2014). Our results also indicate that not all *B. melitensis* isolates from Africa are part of this “Africa” lineage. For example, some samples from Somalia, Kenya, and Egypt appear to be part of the E. Mediterranean clade and Ethiopian and some Egyptian samples are part of the Americas clade. Moreover, Lounes et al. (2014) identified isolates from the W. Mediterranean clade in the Maghreb region of Algeria. Even less is known from sub-Saharan Africa, where brucellosis is widespread among livestock (Mcdermott and Arimi, 2002). Clearly, there is tremendous diversity of *B. melitensis* in Africa, potentially due to many introductions of infected livestock from many regions as well as widespread trade. Additional sampling throughout the African continent is needed to better understand the evolutionary history regarding the origins and spread of *B. melitensis* in the region. Although the African lineage may be more localized than the three widely distributed *B. melitensis* clades, substantial undiscovered diversity likely exists, and as we have discovered in Oman, extends beyond the African continent into new lands that have been connected by trade in camels, goats, and sheep.

## Author contributions

JF, RT, AH, PK, and MS designed the study. MH, AA-R, RT, and MS collected and cultured the samples. JF, FW, BR, KD, and RT analyzed the data. All authors contributed to writing and editing the manuscript.

### Conflict of interest statement

The authors declare that the research was conducted in the absence of any commercial or financial relationships that could be construed as a potential conflict of interest.

## References

[B1] Abbas B. Agab H. (2002). A review of camel brucellosis. Prev. Vet. Med. 55, 47–56. 10.1016/S0167-5877(02)00055-7 12324206

[B2] Afifi S. Earhart K. Azab M. A. Youssef F. G. El Sakka H. Wasfy M.. (2005). Hospital-based surveillance for acute febrile illness in Egypt: a focus on community-acquired bloodstream infections. Am. J. Trop. Med. Hygiene 73, 392–399. 10.4269/ajtmh.2005.73.392 16103611

[B3] Al Dahouk S. Le Fleche P. Nockler K. Jacques I. Grayon M. Scholz H. C.. (2007a). Evaluation of Brucella MLVA typing for human brucellosis. J. Microbiol. Methods 69, 137–145. 10.1016/j.mimet.2006.12.015 17261338

[B4] Al Dahouk S. Neubauer H. Hensel A. Schoneberg I. Nockler K. Alpers K.. (2007b). Changing epidemiology of human brucellosis, Germany, 1962–2005. Emerg. Infect. Dis. 13, 1895–1900. 10.3201/eid1312.070527 18258041 PMC2876757

[B5] Alton G. G. Jones L. M. Angus R. D. Verger J. M. (1988). Bacteriological methods, in Techniques for the Brucellosis Laboratory (Paris: Institut National de la Recherche Agronomique), 13–61.

[B6] Birdsell D. N. Pearson T. Price E. P. Hornstra H. M. Nera R. D. Stone N.. (2012). Melt analysis of mismatch amplification mutation assays (Melt-MAMA): a functional study of a cost-effective SNP genotyping assay in bacterial models. PLoS ONE 7:e32866. 10.1371/journal.pone.0032866 22438886 PMC3306377

[B7] De Massis F. Ancora M. Atzeni M. Rolesu S. Bandino E. Danzetta M. L.. (2015). MLVA as an epidemiological tool to trace back *Brucella melitensis* biovar 1 re-emergence in Italy. Transbound. Emerg. Dis. 62, 463–469. 10.1111/tbed.12397 26194658

[B8] El Tahir Y. E. H. Nair R. R. (2011). Prevalence of brucellosis in the sultanate of Oman with reference to some Middle-East countries. Vet. Res. 4, 71–76. 10.3923/vr.2011.71.76

[B9] Garcell H. G. Garcia E. G. Pueyo P. V. Martín I. R. Arias A. V. Alfonso Serrano R. N. (2016). Outbreaks of brucellosis related to the consumption of unpasteurized camel milk. J. Infect. Public Health 9, 523–527. 10.1016/j.jiph.2015.12.006 26796768

[B10] Garofolo G. Di Giannatale E. De Massis F. Zilli K. Ancora M. Camma C.. (2013). Investigating genetic diversity of *Brucella abortus* and *Brucella melitensis* in Italy with MLVA-16. Infect. Genetics. Evol. 19, 59–70. 10.1016/j.meegid.2013.06.021 23831636

[B11] Georgi E. Walter M. C. Pfalzgraf M.-T. Northoff B. H. Holdt L. M. Scholz H. C.. (2017). Whole genome sequencing of *Brucella melitensis* isolated from 57 patients in Germany reveals high diversity in strains from Middle East. PLoS ONE 12:e0175425. 10.1371/journal.pone.0175425 28388689 PMC5384748

[B12] Gwida M. El-Gohary A. Melzer F. Khan I. Rosler U. Neubauer H. (2012). Brucellosis in camels. Res. Vet. Sci. 92, 351–355. 10.1016/j.rvsc.2011.05.002 21632084

[B13] Gwida M. M. El-Gohary A. H. Melzer F. Tomaso H. Rosler U. Wernery U.. (2011). Comparison of diagnostic tests for the detection of *Brucella* spp. in camel sera. BMC Res. Notes 4:525. 10.1186/1756-0500-4-525 22145943 PMC3284514

[B14] Gyuranecz M. Wernery U. Kreizinger Z. Juhász J. Felde O. Nagy P. (2016). Genotyping of *Brucella melitensis* strains from dromedary camels (*Camelus dromedarius*) from the United Arab Emirates with multiple-locus variable-number tandem repeat analysis. Vet. Microbiol. 186, 8–12. 10.1016/j.vetmic.2016.02.009 27016751

[B15] Hinic V. Brodard I. Thomann A. Cvetnic Z. Makaya P. V. Frey J.. (2008). Novel identification and differentiation of *Brucella melitensis, B. abortus, B. suis, B. ovis, B. canis, and B. neotomae* suitable for both conventional and real-time PCR systems. J. Microbiol. Methods 75, 375–378. 10.1016/j.mimet.2008.07.002 18675856

[B16] Huynh L. Y. Van Ert M. N. Hadfield T. Probert W. S. Bellaire B. H. Dobson M. (2008). Multiple locus variable number tandem repeat (VNTR) analysis (MLVA) of *Brucella* spp. identifies species-specific markers and provides insights into phylogenetic relationships, in NIH: Frontiers in Research, ed St. Georgiev V. (Totowa, NJ: Humana Press), 47–54.

[B17] Jiang H. Fan M. Chen J. Mi J. Yu R. Zhao H.. (2011). MLVA genotyping of Chinese human *Brucella melitensis* biovar 1, 2 and 3 isolates. BMC Microbiol. 11:256. 10.1186/1471-2180-11-256 22108057 PMC3233519

[B18] Kattar M. M. Jaafar R. F. Araj G. F. Le Fleche P. Matar M. G. Rached R. A. (2008). Evaluation of a multilocus variable-number tandem-repeat analysis scheme for typing human *Brucella* isolates in a region of brucellosis endemicity. J. Clin. Microbiol. 45, 3935–3940. 10.1128/JCM.00464-08 PMC2593282 18923007

[B19] Kaufmann A. F. Fox M. D. Boyce J. M. Anderson D. C. Potter M. E. Martone W. J.. (1980). Airborne spread of brucellosis. An. N.Y. Acad. Sci. 353, 105–114. 10.1111/j.1749-6632.1980.tb18912.x 6939379

[B20] Keim P. Van Ert M. N. Pearson T. Vogler A. J. Huynh L. Y. Wagner D. M. (2004). Anthrax molecular epidemiology and forensics: using the appropriate marker for different evolutionary scales. Infect. Genetics Evol. 4, 205–213. 10.1016/j.meegid.2004.02.005 15450200

[B21] Kilic S. Ivanov I. N. Durmaz R. Bayraktar M. R. Ayaslioglu E. Uyanik M. H.. (2011). Multiple-locus variable-number tandem-repeat analysis genotyping of human *Brucella* isolates from Turkey. J. Clin. Microbiol. 49, 3276–3283. 10.1128/JCM.02538-10 21795514 PMC3165627

[B22] Le Fleche P. Jacques I. Grayon M. Al Dahouk S. Bouchon P. Denoeud F.. (2006). Evaluation and selection of tandem repeat loci for a *Brucella* MLVA typing assay. BMC Microbiol. 6:9. 10.1186/1471-2180-6-9 16469109 PMC1513380

[B23] Lounes N. Cherfa M.-A. Le Carrou G. Bouyoucef A. Jay M. Garin-Bastuji B.. (2014). Human brucellosis in Maghreb: existence of a lineage related to socio-historical connections with Europe. PLoS ONE 9:e115319. 10.1371/journal.pone.0115319 25517901 PMC4269447

[B24] Mcdermott J. J. Arimi S. M. (2002). Brucellosis in sub-Saharan Africa: epidemiology, control and impact. Vet. Microbiol. 90, 111–134. 10.1016/S0378-1135(02)00249-3 12414138

[B25] Memish Z. A. Mah M. W. (2001). Brucellosis in laboratory workers at a Saudi Arabian hospital. Am. J. Infect. Control 29, 48–52. 10.1067/mic.2001.111374 11172318

[B26] Moreno E. (2014). Retrospective and prospective perspectives on zoonotic brucellosis. Front. Microbiol. 5:213. 10.3389/fmicb.2014.00213 24860561 PMC4026726

[B27] Musa M. T. Eisa M. Z. El Sanousi E. M. Abdel Wahab M. B. Perrett L. (2008). Brucellosis in camels (*Camelus dromedarius*) in Darfur, Western Sudan. J. Comp. Pathol. 138, 151–155. 10.1016/j.jcpa.2007.10.005 18346482

[B28] Narnaware S. D. Dahiya S. S. Kumar S. Tuteja F. C. Nath K. Patil N. V. (2017). Pathological and diagnostic investigations of abortions and neonatal mortality associated with natural infection of *Brucella abortus* in dromedary camels. Comp. Clin. Path. 26, 79–85. 10.1007/s00580-016-2348-4

[B29] Nascimento M. Sousa A. Ramirez M. Francisco A. P. Carriço J. A. Vaz C. (2017). PHYLOViZ 2.0: Providing scalable data integration and visualization for multiple phylogenetic inference methods. Bioinformatics 33, 128–129. 10.1093/bioinformatics/btw582 27605102

[B30] OIE A. H. S. (2012). Manual of Diagnostic Tests and Vaccines for Terrestrial Animals, Chapter 2.4.3 Bovine brucellosis. Paris: Office International des Epizooties.

[B31] Pappas G. Memish Z. A. (2007). Brucellosis in the Middle East: a persistent medical, socioeconomic and political issue. J. Chemother. 19, 243–248. 10.1179/joc.2007.19.3.243 17594917

[B32] Pappas G. Papadimitriou P. Akritidis N. Christou L. Tsianos E. V. (2006). The new global map of human brucellosis. Lancet Infect. Dis. 6, 91–99. 10.1016/S1473-3099(06)70382-6 16439329

[B33] Radwan A. I. Bekairi S. I. Mukayel A. A. Albokmy A. M. Prasad P. V. S. Azar F. N.. (1995). Control of *Brucella melitensis* infection in a large camel herd in Saudi Arabia using antibiotherapy and vaccination with Rev. 1 vaccine. Rev. Sci. Tech. 14, 719–732. 10.20506/rst.14.3.860 8593404

[B34] Refai M. (2002). Incidence and control of brucellosis in the Near East region. Vet. Microbiol. 90, 81–110. 10.1016/S0378-1135(02)00248-1 12414137

[B35] Sahl J. W. Lemmer D. Travis J. Schupp J. Gillece J. Aziz M. (2016). The Northern Arizona SNP Pipeline (NASP): accurate, flexible, and rapid identification of SNPs in WGS datasets. bioRxiv. 10.1101/037267 PMC5320593 28348869

[B36] Scrimgeour E. M. Mehta F. R. Suleiman A. J. (1999). Infectious and tropical diseases in Oman: a review. Am. J. Trop. Med. Hyg. 61, 920–925. 10.4269/ajtmh.1999.61.920 10674671

[B37] Seleem M. N. Boyle S. M. Sriranganathan N. (2010). Brucellosis: a re-emerging zoonosis. Vet. Microbiol. 140, 392–398. 10.1016/j.vetmic.2009.06.021 19604656

[B38] Shimol S. B. Dukhan L. Belmaker I. Bardenstein S. Sibirsky D. Barrett C.. (2012). Human brucellosis outbreak acquired through camel milk ingestion in southern Israel. Isr. Med. Assoc. J. 14, 475–478. 22977965

[B39] Sprague L. D. Al-Dahouk S. Neubauer H. (2012). A review on camel brucellosis: a zoonosis sustained by ignorance and indifference. Pathog. Glob. Health 106, 144–149. 10.1179/2047773212Y.0000000020 23265371 PMC4001573

[B40] Tabbara K. F. (1993). Brucellosis: a model for eradication of endemic diseases from the Arabian peninsula. Ann. Saudi Med. 13, 1–2. 10.5144/0256-4947.1993.1 17587973

[B41] Tan K.-K. Tan Y.-C. Chang L.-Y. Lee K. W. Nore S. S. Yee W. -Y.. (2015). Full genome SNP-based phylogenetic analysis reveals the origin and global spread of *Brucella melitensis*. BMC Genomics 16:93. 10.1186/s12864-015-1294-x 25888205 PMC4409723

[B42] Tay B. Y. Ahmad N. Hashim R. Mohamed Zahidi J. A. Thong K. L. Koh X. P.. (2015). Multiple-locus variable-number tandem-repeat analysis (MLVA) genotyping of human *Brucella* isolates in Malaysia. BMC Infect. Dis. 15:220. 10.1186/s12879-015-0958-0 26033227 PMC4450988

[B43] Tiller R. V. De B. K. Boshra M. Huynh L. Y. Van Ert M. N. Wagner D. M.. (2009). Comparison of two multiple-locus variable-number tandem-repeat analysis methods for molecular strain typing of human *Brucella melitensis* isolates from the Middle East. J. Clin. Microbiol. 47, 2226–2231. 10.1128/JCM.02362-08 19439543 PMC2708484

[B44] Vassallo D. J. (1996). The saga of brucellosis: controversy over credit for linking Malta fever with goats' milk. Lancet 348, 804–808. 10.1016/S0140-6736(96)05470-0 8813991

[B45] Wernery U. (2014). Camelid brucellosis: a review. Rev. Sci. Tech. 33, 839–857. 10.20506/rst.33.3.2322 25812208

[B46] Whatmore A. M. (2009). Current understanding of the genetic diversity of *Brucella*, an expanding genus of zoonotic pathogens. Infect. Genet. Evol. 9, 1168–1184. 10.1016/j.meegid.2009.07.001 19628055

[B47] Whatmore A. M. Koylass M. S. Muchowski J. Edwards-Smallbone J. Gopaul K. K. Perrett L. L. (2016). Extended multilocus sequence analysis to describe the global population structure of the genus *Brucella*: phylogeography and relationship to biovars. Front. Microbiol. 7:2049. 10.3389/fmicb.2016.02049 28066370 PMC5174110

[B48] Wilson R. T. (1984). The Camel. New York, NY: Longman.

[B49] Wyatt H. V. (2005). How Themistocles Zammit found Malta Fever (brucellosis) to be transmitted by the milk of goats. J. R. Soc. Med. 98, 451–454. 10.1177/014107680509801009 16199812 PMC1240100

